# Genetic analysis of morphological traits in a new, versatile, rapid-cycling *Brassica rapa* recombinant inbred line population

**DOI:** 10.3389/fpls.2012.00183

**Published:** 2012-08-16

**Authors:** Hedayat Bagheri, Mohamed El-Soda, Inge van Oorschot, Corrie Hanhart, Guusje Bonnema, Tanja Jansen-van den Bosch, Rolf Mank, Joost J. B. Keurentjes, Lin Meng, Jian Wu, Maarten Koornneef, Mark G. M. Aarts

**Affiliations:** ^1^Laboratory of Genetics, Wageningen UniversityWageningen, Netherlands; ^2^Bu-Ali Sina UniversityHamedan, Iran; ^3^Faculty of Agriculture, Department of Genetics, Cairo UniversityGiza, Egypt; ^4^Laboratory of Plant Breeding, Wageningen UniversityWageningen, Netherlands; ^5^Keygene N. V.Wageningen, Netherlands; ^6^Institute of Vegetables and Flowers, Chinese Academy of Agricultural SciencesBeijing, China; ^7^Max Planck Institute for Plant Breeding ResearchCologne, Germany

**Keywords:** *Brassica rapa*, recombinant inbred line population, QTL analysis, plant breeding

## Abstract

A recombinant inbred line (RIL) population was produced based on a wide cross between the rapid-cycling and self-compatible genotypes L58, a Caixin vegetable type, and R-o-18, a yellow sarson oil type. A linkage map based on 160 F7 lines was constructed using 100 Single nucleotide polymorphisms (SNPs), 130 AFLP®, 27 InDel, and 13 publicly available SSR markers. The map covers a total length of 1150 centiMorgan (cM) with an average resolution of 4.3 cM/marker. To demonstrate the versatility of this new population, 17 traits, related to plant architecture and seed characteristics, were subjected to quantitative trait loci (QTL) analysis. A total of 47 QTLs were detected, each explaining between 6 and 54% of the total phenotypic variance for the concerned trait. The genetic analysis shows that this population is a useful new tool for analyzing genetic variation for interesting traits in *B. rapa*, and for further exploitation of the recent availability of the *B. rapa* whole genome sequence for gene cloning and gene function analysis.

## Introduction

*Brassica rapa* is an important, widely cultivated crop, with various forms or “morphotypes”, such as leafy vegetables, turnips, and oilseed rape (Zhao et al., 2005). While the use of *B. rapa* as an oilseed crop is relatively modest, it is important as one of the parents of *Brassica napus*, the most important oilseed crop. After the oil has been extracted from the seeds, the remaining seed components (meal) are of economic interest for feeding animals. It has been known for some time that breeding for yellow seed color is advantageous for meal quality in *B. napus*, because yellow-seeded genotypes have a thinner seed coat associated with a higher protein content and less non-energetic and anti-nutritive fiber components (Liu et al., 2012). Therefore, breeding programs aiming at combining yellow seed color with yield associated traits such as seed number, seed size, number of siliques per plant, pod shattering, carpel number, and vivipary, have been developed in *B. napus* through interspecific crosses with yellow-seeded *Brassica* species (Tang et al., 1997; Badani et al., 2006; Wittkop et al., 2009; Liu et al., 2012). A problem is that the expression of the yellow color, at least in *B. napus*, is highly dependent on environmental factors (Liu et al., 2012).

Pod shattering, caused by carpel abscission, is an undesirable characteristic in crop breeding as it decreases the yield due to seed loss during harvesting. For *B. napus* the seed yield loss can be as much as 20% of the harvest (Price et al., 1996). The absence of embryonic dormancy during seed development, which prevents seeds to germinate prematurely on the mother plant, can be expressed as vivipary. While this is more commonly observed in cereals, it can also be found in oilseed rape, leading to large economic losses due to significant reduction in seed quality. Resistance to vivipary is therefore a very favorable trait in breeding programs (Zhang et al., 2008). Next to seed related traits, plant height (Ph), branch number (Bn), and leaf number at first flower opening are factors contributing to Brassica plant architecture that differ considerably between genotypes. Plant architecture is of major agronomic importance and has a strong effect on the suitability of a plant species for cultivation, as it affects plant yield and harvest efficiency (Reinhardt and Kuhlemeier, 2002).

With the smallest genome size in the *Brassica* genus, the rapid life cycle of some of its genotypes, and the relatively close relationship to the model plant species *Arabidopsis thaliana*, *B. rapa* is considered to be one of the model dicot crops for genetic studies (Wang et al., 2011a). These studies require “immortal” mapping populations, i.e., populations that can be easily propagated through seed without altering their genotypes, as indispensable tools in identifying quantitative trait loci (QTLs) underlying traits of interest (Koornneef et al., 2004). Doubled haploid (DH) populations are the most commonly used type of immortal mapping populations for *Brassica* species (Pink et al., 2008). However, the poor response of many *B. rapa* genotypes to DH induction (Kole et al., 1997) together with the high degree of segregation distortion often observed in DH populations (Voorrips et al., 1997), limits this use. Instead, when using self-compatible genotypes with short generation times it is feasible to develop Recombinant Inbred Line (RIL) populations through sexual propagation. In this study two *B. rapa* genotypes, corresponding to two distinct morphotypes, the leafy vegetable Cai Xin accession L58, of Chinese ancestry, and the yellow sarson oil seed DH line R-o-18, of Indian ancestry, were crossed to generate a RIL population. Both parents are early flowering and self-compatible, which facilitates rapid propagation and the ability to maintain the RILs through single seed descent.

Genetic linkage maps are required to properly query DH or RIL populations for the identification of the chromosomal regions or QTLs that harbor the genes controlling important agronomic traits. Single nucleotide polymorphisms (SNPs) represent the most abundant and common type of genetic polymorphisms that can be readily converted into genetic markers for marker assisted selection. Large-scale SNP discovery projects, using high-throughput sequencing techniques, have become a powerful complement to the standard genetic mapping procedures, and the use of resulting markers greatly improves the linkage maps of diploid crops. The Illumina GoldenGate assay is an efficient SNP genotyping tool that has been used already for soybean, tetraploid and hexaploid wheat lines, and maize (Hyten et al., 2008; Akhunov et al., 2009; Yan et al., 2010). Currently, SNP genotyping is replacing the use of the AFLP technology, which has previously been very useful for analyzing genetic diversity and relationships in many plant species, including *B. rapa*, identifying a large number of polymorphic loci (Zhao et al., 2005).

This paper describes the generation and genetic mapping of a large, versatile, rapid-cycling *B. rapa* RIL population dedicated for QTL analysis. As an illustration of the potential importance of this population, we used it to identify 47 QTLs, responsible for most of the observed morphological variation in 17 different traits.

## Materials and methods

### Plant growth and generation of the RIL population

The two parental genotypes L58 and R-o-18 were crossed reciprocally and from each of the two F1 offspring, one plant was randomly selected to be propagated by subsequent generations of self-fertilization using a single-seed-descent approach, aimed at minimizing any bias in selecting plants. The seeds of L58 (*B. rapa* ssp. *parachinensis*) were provided by Dr. Xiaowu Wang from the Institute for Vegetables and Flowers of the Chinese Academy of Agricultural Sciences, Beijing, China; and seeds of R-o-18 (*B. rapa* var. *trilocularis*) were obtained from Dr. Lars Østergaard, John Innes Centre, Norwich, UK. One of the two F1 combinations, L58 (♀) × R-o-18 (♂), was propagated until the F7 generation, the other remained at F5 and could be used for future fine-mapping studies. All generations were grown between April 2007 and June 2009 with four replications in a fully randomised design. Individual plants were grown in 19-cm diameter black plastic pots filled with a potting soil consisting of prefertilized peat, obtained from “Lentse potgrond” (www.lentsepotgrond.nl), in a temperature-controlled greenhouse at 21°C with artificial long day light (16 h). No cold treatment or vernalization was applied for germination or flowering respectively. For every generation, the first flower appeared about four weeks after germination in the early flowering lines. The inflorescences were covered with perforated plastic bags to prevent cross-pollination by insects. In case of poor seed set, hand pollinations were performed. The 160 F7 RILs were multiplied in the same conditions, ensuring homogeneous material for genetic studies.

### DNA extraction and genotyping

DNA was extracted from frozen F7 leaves according to a modified CTAB procedure (Beek et al., 1992). The DNA was amplified with the Genomiphi-kit (*Illustra*™ *GenomiPhi*™ *V2 DNA Amplification Kit*, GE Healthcare UK) to be suitable for GoldenGate assay analysis (Akhunov et al., 2009). For SNP discovery, two *B. rapa* lines (Kenshin and Chiifu) were compared using CRoPS®-technology (van Orsouw et al., 2007) to reveal more than 1300 putative SNPs. The SNP-harboring sequences were processed with the Illumina Assay Design Tool (ADT) by Illumina (www.illumina.com). A total of 384 SNPs were selected, all having ADT scores above 0.6. 100–500 ng of genomic DNA (GenomiPhi) per plant was used for Illumina SNP genotyping at Keygene N.V. using the Illumina BeadXpress™ platform and the GoldenGate Assay. Part of the DNA was used for SSR or AFLP detection as described by Choi et al. (2007) and Vos et al. (1995), respectively. Pre-amplification and selective amplification for AFLP analysis were carried out as described by Zhao et al. (2005). For selective amplification seven combinations of EM (*Eco*RI*/Mse*I) primers (E34M15, E34M16, E37M32, E37M49, E37M56, E40M38, and E40M51) and four combinations of PM (P*st*I/*Mse*I) primers (P23M48, P23M50, P21M47, and P23M47) were used. The *Pst* I and *Eco*RI primers were labeled with IRD-700 at their 5′ ends (Zhao et al., 2005). The reaction product of selective amplification was mixed with an equal volume of formamide-loading buffer, denatured for 5 min at 94°C, cooled on ice and run on a 5.5% denaturing polyacrylamide gel using the LI-COR system 4200 DNA sequencer (Li-Cor, Lincoln, Neb.) (Myburg et al., 2001). The AFLP gel images were analyzed by the AFLP-Quantar Pro software. All distinguishable bands ranging from 50 to 500 bp were used in the data analysis. AFLP bands were scored as 1 or 0 for presence or absence of the band, respectively. All weak and ambiguous bands were scored as “unknown”. In addition, 36 public SSR primer pairs (Choi et al., 2007) were used to screen for polymorphisms using the same LI-COR system to run a 5.5% denaturing polyacrylamide gel. Furthermore, 27 polymorphic InDel markers, based on DNA resequencing information of two parental lines of a DH population, which was used to construct a *B. rapa* reference map for pseudochromosome sequence assembly, were screened as described by (Wang et al., 2011b).

### Construction of a genetic linkage map and QTL analysis

The genetic map was constructed using JoinMap 4.0 (www.kyazma.nl). Monomorphic markers, markers with a high number of unknown scores and markers with more than 75% allele skewedness toward either A or B were removed. Recombination frequencies were converted to centiMorgan (cM) distances using Haldane's mapping function. SNP markers positions were confirmed by comparing their primer sequences with the *B. rapa* genome using the *Brassica* database (BRAD) (brassicadb.org) of *Brassica* crops whole genome sequence and genetics data (Cheng et al., 2011). It contains the complete *Brassica* A genome sequence from the reference *B. rapa* genotype Chiifu-401-42 (Wang et al., 2011a). InDel markers were compared to the reference map (Choi et al., 2007; Wang et al., 2011b), which was previously used for chromosome alignment.

MAPQTL 6.0 (www.kyazma.nl) was used for QTL analysis. First, the interval mapping procedure was performed to detect major QTLs. For each trait a 1000 X permutation test was performed to calculate the LOD threshold corresponding to a genome-wide false discovery rate of 5% (*P* < 0.05). Markers with LOD scores equal to or exceeding the threshold were used as cofactors in multiple-QTL-model (MQM) mapping. If new QTLs were detected, the linked markers were added to the cofactor list and the MQM analysis was repeated. If the LOD value of a marker dropped below the threshold in the new model, it was removed from the cofactor list and the MQM analysis was rerun. This procedure was repeated until the cofactor list became stable. The final LOD score for each trait was determined by restricted MQM (rMQM) mapping. In some cases, rMQM mapping showed that some cofactors should be on the same linkage group, but at slightly different positions. In that case, the new marker was selected as a cofactor and the whole procedure was repeated. The linkage map was visualized using Mapchart (Voorrips, 2002).

### Trait measurement

**Figure 1:**
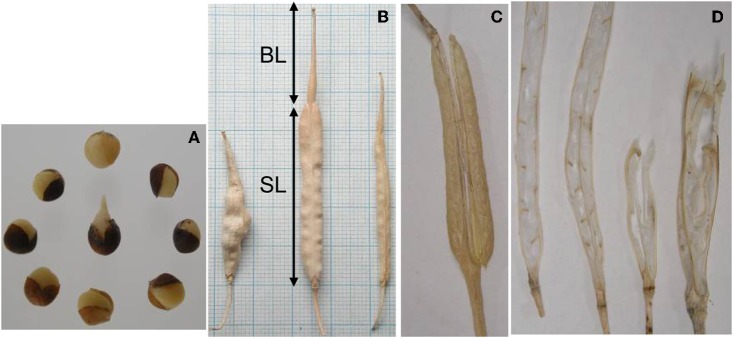
**Phenotyping of RIL population of *B. rapa* L58 × R-o-18. (A)** Seed vivipary; i.e., premature germination of seeds still in the silique, or just after harvesting, **(B)** silique length (SL) and silique beak length (BL), **(C)** pod shattering, corresponding to the fraction of opened siliques at harvesting, **(D)** carpel number, with the left two siliques having two carpels and the two on the right having three.

The 160 RILs (four replicate plants) and both parents (five replicate plants) were phenotyped for 17 traits. These traits are categorized into two main groups. Seed related traits, including seed color, seed weight, seed oil, seed germination, and seed vivipary; and morphological traits, including flowering time (Ft), total height, Ph until the first flower, Bn, silique length (Sil), silique beak length (Bl), silique number (Sin), number of seeds per silique (Nsps), carpel number, pod shattering, total leaf number (Tln), and leaf number until the first flower (Lnf). Seed color of fully mature F8 seeds was visually scored and ranked into nine different classes ranging from yellow (1) to black (9). Seed germination data were obtained by sowing 30 seeds of each line and scoring the percentage of germination 15 h after sowing. The seeds were sterilized in 2% sodium hypochlorite for 2 min. After rinsing 2 times with sterile distilled water, they were sown in two rows of 15 seeds on square plates containing 50 ml of half MS medium +1% agar. The plates were placed vertically in a 25°C growth chamber with a 16/8 h light/dark photoperiod. Sil and Nsps were averaged from three ripe siliques. Seed vivipary was scored as either 0 (no vivipary), 0.5 (medium), or 1 (high) based on visual estimation of the number of seeds with radicles when harvested. Shattering was scored at harvesting time as either 0 (no open siliques), 0.5 (few open siliques), or 1 (many open siliques) (Figure 1). Seed oil was extracted by a crude method of hexane extraction, grinding 10 weighed F7 seeds of each line in 650 μl of hexane, shaking the mix for 2 min followed by 1 min of centrifugation at 14,000 rpm in an Eppendorf microfuge. 600 μl of supernatant was transferred to a new tube and left overnight in the fume hood to evaporate the hexane. The oil content was determined in mg oil per mg seed (Goossens et al., 1999). All traits were measured for each of the four replicate plants, and the average values were used for mapping, except for seed color, seed germination, and seed oil content, for which only one replication could be measured. The heritability was calculated as the ratio between the genetic variation (Vg), i.e., variance between the average values of all RILs, and the total variation (Vt), with Vt = Vg + Ve, where Ve is the environmental variation, i.e., variance between the replications of all lines. All statistical analysis was performed in SPSS 19.

## Results

### Genotyping and construction of the linkage map for the RIL population

**Figure 2:**
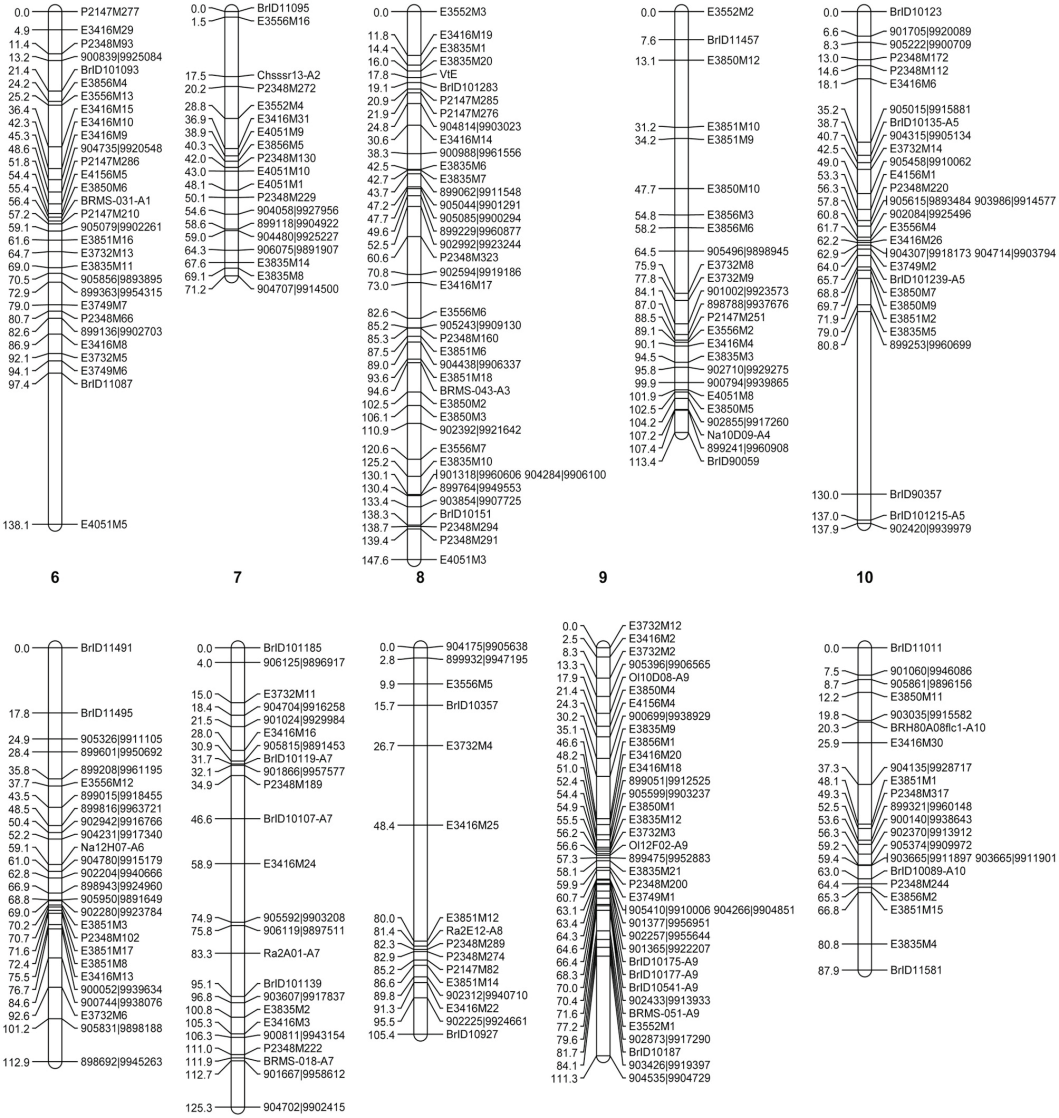
**Genetic linkage map of the *B. rapa* L58 × R-o-18 RIL population, showing the positions of 270 markers (100 SNP, 130 AFLP, 27 InDel, and 13 SSR markers) distributed over 10 linkage groups corresponding to the 10 chromosomes of the *Brassica* A genome.** Markers labeled with [6 digits|7 digits] are SNPs, markers labeled “E … M..” or “P … M..” are respectively EcoRI/MseI or PstI/MseI generated AFLPs, markers labeled “BrID ….” are InDels and the remaining markers are SSRs.

The availability of the complete genome sequence of *B. rapa* (Wang et al., 2011a) and the genome analysis tools provided in the BRAD database (Cheng et al., 2011), were critical for constructing a reliable genetic map of the L58 × R-o-18 RIL population suitable for QTL mapping. Out of the 384 SNPs that could be queried by the Brassica GoldenGate assay we used, 120 SNPs were polymorphic between the parents, of which 100 provided unambiguous genotype calls for mapping. Based on the sequence of the SNP primers, the position of the 100 mapped SNP markers could be linked to their sequence position on the *B. rapa* genome, thus confirming the mapping results and providing anchoring points for chromosome number assignment and proper orientation of the chromosomal linkage maps with the genome sequence. The same was done for the SSR markers previously used to create the *B. rapa* reference linkage map (Choi et al., 2007). In total 94 InDel markers were screened, from which 27 showed polymorphism between the two parental lines. Seven of these polymorphic markers have been mapped on the reference map used for *B. rapa* pseudochromosome assembly (Wang et al., 2011b), while the other markers were assigned to the chromosomes according to the position of their corresponding sequence scaffolds. The final linkage map was constructed for the L58 × R-o-18 F7 RIL population using 100 SNP, 130 AFLP, 27 InDel, and 13 SSR markers. It covers a total length of 1150 cM with an average resolution of 4.3 cM per marker (Figure 2).

### Phenotyping the RIL population

**Figure 3:**
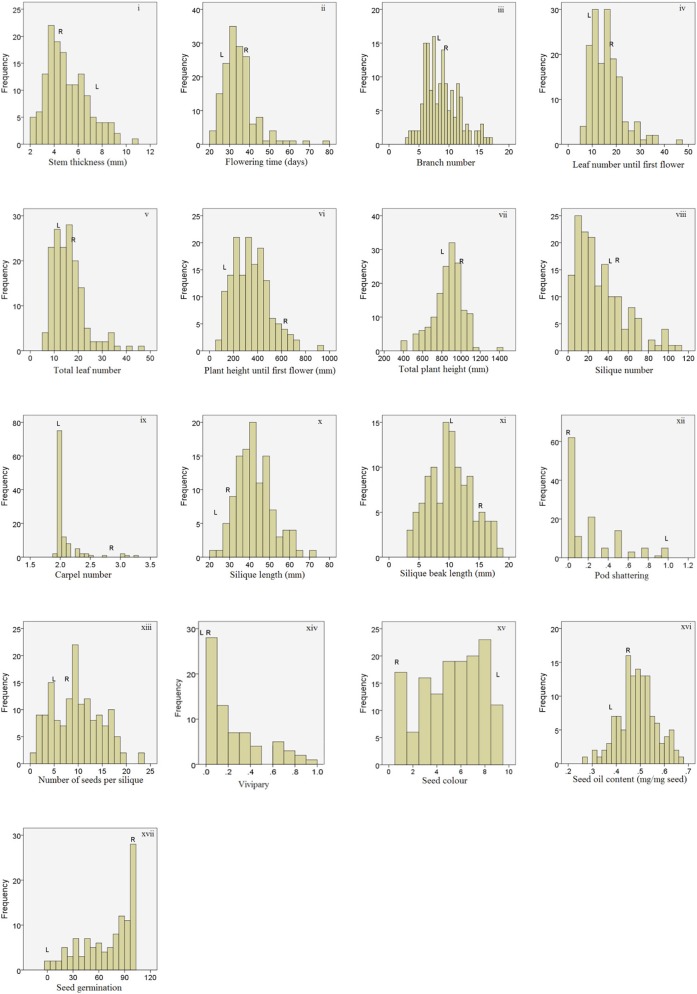
**Frequency distributions of non-normalized data of the reported traits for the L58 × R-o-18 RIL population.** The vertical axes indicate the number of lines per trait value class and the horizontal axes indicate the different trait value classes. The parental values (indicated with L and R) are the mean of five replicates.

A total of 17 traits were analyzed for the F7 RIL population. Figure 3 shows the frequency distributions of the measured traits over the whole population. Transgression beyond the parental line values was observed for most of the traits except seed color, pod shattering, seed germination, and vivipary. Broad sense heritabilities ranged from 0.35, for stem thickness, to 0.92, for Ft (Table 1). Heritabilities could not be determined for seed color, seed germination, and seed oil content, as for these traits only one replication could be measured. Correlation analysis of all measured traits (Table 2) showed that Ft was highly positively correlated with Tln and Lnf. Sin, Nsps, pod shattering, and Sil were also positively correlated. In general, plants with more siliques had longer siliques with more seeds and higher seed oil content, all contributing to traits favored for oil seed rape.

**Table 1:** **Phenotype data for both parental lines (L58 and R-o-18) and the RIL population, for the 17 analyzed traits**.

**Trait (unit)**	**Abbreviation**	**Parental lines**			**RIL population**			**Mean**	**SD**	**h^2^**
		**L58**	**R-o-18**	**%**	**Min**	**Max**	**Range**			
Stem thickness (mm)	*St*	7.5	4.5	60.0	2.0	10.8	8.7	5.1	1.7	0.35
Flowering time (days)	*Ft*	28.8	38.4	133.6	22.0	79.8	57.8	34.6	8.2	0.92
Branch number	*Bn*	8.4	8.9	105.9	3.0	17.0	14.0	8.7	2.9	0.69
Leaf number until first flower	*Lnf*	11.8	17.8	151.8	5.0	46.0	41.0	15.9	6.6	0.89
Total leaf number	*Tln*	13.9	17.8	128.1	5.5	46.8	41.3	15.9	6.9	0.89
Plant height until first flower (mm)	*Ph*	171	658	384	88	943	855	344	154	0.81
Total plant height (mm)	*Tph*	878	1078	123	398	1403	1005	864	156	0.76
Silique number	*Sin*	46.5	48.8	104.8	0.0	108.5	108.5	32.3	24.1	0.73
Carpel number	*Cn*	2.0	2.8	141.3	1.9	3.3	1.3	2.1	0.2	0.50
Silique length (mm)	*Sil*	22.9	34.6	151.3	23.3	70.9	47.6	42.5	9.1	0.78
Silique beak length (mm)	*Bl*	10.2	14.8	145.4	3.7	18.5	14.8	10.3	3.6	0.71
Pod shattering	*Sh*	1.0	0.0	0.0	0.0	1.0	1.0	0.2	0.3	0.50
Number of seeds per silique	*Nsps*	6.2	8.1	131.1	1.0	22.9	21.9	9.7	5.0	0.61
Vivipary	*Vi*	0.0	0.0	−	0.0	1.0	1.0	0.2	0.3	0.68
Seed color	*Sc*	9.0	1.0	11.1	1.0	9.0	8.0	5.3	2.5	−
Seed oil content (mg oil/mg seed)	*So*	0.4	0.4	112.8	0.3	0.7	0.4	0.5	0.1	−
Seed germination	*Sg*	0.0	100.0	−	0.0	100.0	100.0	68.1	29.8	−

“%” indicates the relative performance of R-o-18 compared to L58. “Min” and “Max” indicate the values of the RIL with respectively the lowest of the highest value, while “Range” indicates the difference between these values. “Mean” is the average value for all RIL lines, with standard deviation (SD), and h^2^ is broad sense heritability. For all traits four replicate samples were measured, except for seed color, seed germination, and seed oil content, for which only one sample could be measured. All 160 lines have been scored.

**Table 2:** **Pearson correlations for the analyzed traits of the L58 × R-o-18 RIL population**.

**Trait**	***St***	***Ft***	***Bn***	***Lnf***	***Tln***	***Ph***	***Tph***	***Sin***	***Cn***	***Sil***	***Bl***	***Sh***	***Nsps***	***Vi***	***Sc***	**So**	**Sg**
*St*	1	–	–	–	–	–	–	–	–	–	–	–	–	–	–	–	–
*Ft*	0.571^**^	1	–	–	–	–	–	–	–	–	–	–	–	–	–	–	–
*Bn*	0.376^**^	0.382^**^	1	–	–	–	–	–	–	–	–	–	–	–	–	–	–
*Lnf*	0.562^**^	0.847^**^	0.563^**^	1	–	–	–	–	–	–	–	–	–	–	–	–	–
*Tln*	0.566^**^	0.861^**^	0.557^**^	0.988^**^	1	–	–	–	–	–	–	–	–	–	–	–	–
*Ph*	0.183^*^	0.275^**^	0.354^**^	0.315^**^	0.289^**^	1	–	–	–	–	–	–	–	–	–	–	–
*Tph*	0.252^**^	−0.014	0.157^*^	0.016	0.012	0.629^**^	1	–	–	–	–	–	–	–	–	–	–
*Sin*	0.079	0.084	0.025	0.067	0.074	−0.109	−0.084	1	–	–	–	–	–	–	–	–	–
*Cn*	0.127	0.064	0.032	0.179	0.157	0.174	0.127	−0.025	1	–	–	–	–	–	–	–	–
*Sil*	−0.191^*^	−0.108	−0.078	−0.105	−0.123	0.196^*^	0.107	0.184	−0.181	1	–	–	–	–	–	–	–
*Bl*	−0.137	−0.003	−0.053	−0.028	−0.021	0.175	0.081	0.198^*^	−0.006	0.680^**^	1	–	–	–	–	–	–
*Sh*	0.053	0.072	0.021	0.033	0.054	−0.148	−0.028	0.419^**^	0.045	0.047	0.186	1	–	–	–	–	–
*Nsps*	0.249^**^	0.171^*^	0.120	0.139	0.143	0.085	0.097	0.367^**^	0.166	0.151	0.075	0.355^**^	1	–	–	–	–
*Vi*	−0.344^**^	−0.369^**^	−0.256^*^	−0.392^**^	−0.388^**^	−0.188	0.018	0.147	−0.150	−0.001	0.103	−0.015	−0.305^*^	1	–	–	–
*Sc*	−0.094	−0.075	−0.007	−0.121	−0.120	0.012	0.004	0.060	0.142	−0.166	0.021	0.238^**^	0.170^*^	−0.095	1	–	–
*So*	0.205^*^	0.285^**^	0.076	0.222^*^	0.221^*^	0.010	−0.112	0.345^**^	0.020	−0.040	−0.048	0.113	0.516^**^	−0.228	0.179^*^	1	–
*Sg*	0.017	−0.105	0.091	−0.104	−0.080	0.052	−0.020	0.032	0.024	−0.045	−0.154	−0.123	0.007	0.173	−0.127	−0.004	1

St, stem thickness; Ft, flowering time; Bn, branch number; Lnf, leaf number until first flower; Tln, total leaf number; Ph, plant height until first flower; Tph, total plant height; Sin, silique number; Cn, carpel number; Sil: silique length; Bl, silique beak length; Sh, Pod shattering; Nsps, number of seed per silique; Vi, vivipary; Sc, seed color; So, seed oil content; Sg, seed germination.

** means significant at P ≤ 0.01

* significant at P ≤ 0.05.

### QTL analysis

**Figure 4:**
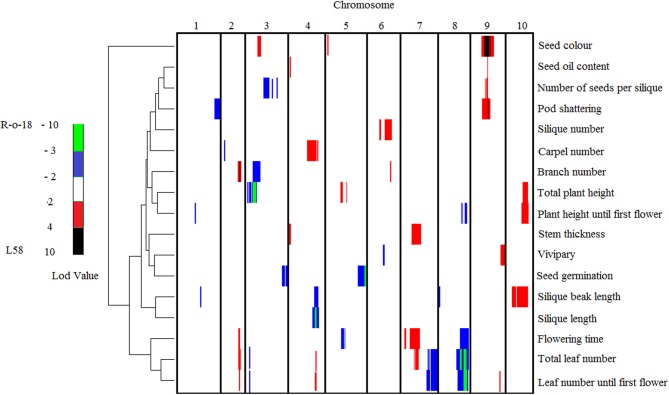
**A clustered heat map showing the LOD profiles of the measured traits.** Columns indicate the 10 chromosomes in centimorgans, ascending from the left to right; rows indicate individual trait LOD profiles. A color scale is used to indicate the QTL significance corresponding to the LOD score. Positive values (red and black) represent a positive effect of the trait by the L58 allele, negative values (blue and green) represent a positive effect on the trait by the R-o-18 allele. The width of a bar indicates the significance interval of the QTL that was calculated by rMQM in MAPQTL 6. Hierarchical clustering, shown on the left, reflects the correlation between traits based on the QTL profiles.

In total 47 QTLs were mapped for the 17 analyzed traits (Table 3 and Figure 4). Seed color was a very prominent phenotype segregating in the population. A major QTL for seed color (*Sc1*) was mapped to chromosome A9 with a LOD score of 30.8 and explaining 53.7% of the total seed color variance. This region on A9 appears to be rich in genetic variation, with several other QTLs co-located with *Sc1*, which are loci for pod shattering (*Sh*), number of seeds per silique (*Nsps1*), and seed oil (*So*). The *Sh* QTL also explains a large portion, 18%, of the phenotypic variance. Another QTL for seed color (*Sc2*), with a LOD score of 12.1, was mapped to chromosome A3, accounting for 15% explained variance. Variation in vivipary (Vi) was explained by two loci, one locus on A9 (*Vi1*), with 20% explained variance, and another on A6 (*Vi2*) that explains 13% of the variance. The carpel number QTL (*Cn1*) co-localized with the *Sil* QTL on A4, each explaining 15%, respectively 17% of the variance. This region also harbors one of the Bl QTLs (*Bl3*). As can be seen from Figure 1, these traits appear to be pleiotropic effects of the same locus, as the increase in carpel number often corresponds with malformed, shorter siliques with shorter beaks. Pleiotropy is also the likely cause of the co-localization of Ft QTLs *Ft3, Ft4*, and *Ft5* with QTLs for Tln (*Tln1, Tln2, Tln4*) and Lnf (*Lnf1, Lnf3, Lnf4*) on respectively A2, A7, and A8. Tln and Lnf share four of the six QTLs found for these traits, in line with the correlation found between them. The locus on A8 also seems to account for variation for Bn, harboring the major Bn QTL (*Bn1*). Ph until the first flower and total Ph (Tph) also share one common QTL, on A10 (*Ph1* and *Tph2*).

**Table 3:** **QTLs detected for the analyzed traits in the L58 × R-o-18 RIL population**.

**Trait**	**QTL**	**Linkage group**	**LOD threshold**	**LOD**	**Position of LOD peak (cM)**	**R^2^**	**Effect**
Stem thickness	*St1*	A7	3	4.7	58.9	13.1	1.3
	*St2*	A4	–	2.8	7.6	8	1.1
Flowering time	*Ft1*	A7	2.8	5.4	34.9	11.2	5.5
	*Ft2*	A5	–	4.9	60.8	9	−5.4
	*Ft3*	A8	–	4.9	85.2	9	−5.1
	*Ft4*	A2	–	3.7	64.3	6.6	4.2
	*Ft5*	A7	–	3.1	106.3	6.5	−4.5
Branch number	*Bn1*	A2	3.1	5.5	64.3	13.1	2.1
	*Bn2*	A3	–	3.2	38.3	7.4	−1.7
	*Bn3*	A6	–	2.8	76.7	6.4	1.5
Leaf number until first flower	*Lnf1*	A8	2.9	8.8	91.3	15	−5.3
	*Lnf2*	A4	–	4.9	90.1	8	3.8
	*Lnf3*	A7	–	4.8	96.7	8	−3.9
	*Lnf4*	A2	–	3.9	64.3	6.2	3.4
	*Lnf5*	A5	–	3.2	35.2	5	−3.1
	*Lnf6*	A9	–	2.9	79.6	4.5	−2.8
Total leaf number	*Tln1*	A8	2.9	8.3	91.3	14	−5.3
	*Tln2*	A7	–	5.7	106.4	10.6	−4.8
	*Tln3*	A7	–	4.1	58.9	7.6	4.1
	*Tln4*	A2	–	4.6	64.3	7.3	3.8
	*Tln5*	A4	–	3.3	77.8	5.1	3.2
	*Tln6*	A3	–	3	38.3	4.6	−3.3
Plant height until first flower	*Ph1*	A10	2.8	4	59.2	9.3	95.4
	*Ph2*	A8	–	3.6	81.4	8.3	−90.2
	*Ph3*	A1	–	2.9	55.5	6.6	−85.2
Total plant height	*Tph1*	A3	3	5	38.3	11.3	−112.3
	*Tph2*	A10	–	4.1	64.4	9.5	96.1
	*Tph3*	A5	–	3.2	69.7	7.1	87.2
Silique number	*Sin*	A6	3	4	72.4	11	16.0
Carpel number	*Cn1*	A4	2.7	4.4	84.1	15.2	0.2
	*Cn2*	A2	–	2.5	17.5	8.5	−0.1
Silique length	*Sil*	A4	3	5	90.1	18.6	−8.1
Silique beak length	*Bl1*	A10	3	5.7	53.6	14.4	2.8
	*Bl2*	A8	–	4.4	0	11	−2.4
	*Bl3*	A4	–	4.1	90.1	10	−2.3
	*Bl4*	A1	–	3	72.9	7.2	−2.0
Pod shattering	*Sh*	A9	3	5.5	60	18	0.2
Number of seeds per silique	*Nsps1*	A9	3	3.5	58.1	10	3.1
	*Nsps2*	A3	–	2.7	82.6	7.2	−2.7
Vivipary	*Vi1*	A9	3	3.9	111.3	20.5	0.3
	*Vi2*	A6	–	2.6	61.1	13.3	−0.2
Seed color	*Sc1*	A9	3	30.8	56.6	53.7	3.7
	*Sc2*	A3	–	12.1	52.5	15	2.1
Seed oil content	*So*	A9	3	3	58.2	9.1	0.1
Seed germination	*Sg1*	A5	3.1	4.1	137	14.4	−23.2
	*Sg2*	A3	–	3.1	147.6	10.5	−22.1

Per trait, QTLs are numbered according to decreasing LOD score (LOD). LOD thresholds are calculated per trait based on 1000 permutation tests and an experimental error rate of P < 0.05. R^2^ is the percentage of total phenotypic variance explained by each QTL. For each QTL, the allelic effect is calculated as μA-μB (μ = mean), where A and B are RILs carrying L58, respectively, R-o-18 alleles at the QTL.

## Discussion

The L-58 × R-o-18 population is a new RIL population, designed for general QTL mapping studies. The parents of this population were selected for a number of reasons. Rapid-cycling and self-compatibility were two important reasons, as these would permit the rapid construction of the population and easy maintenance through single-seed-descent propagation. These are also the reasons that both parents are more and more used as reference genotypes, expanding their use for other purposes, such as the generation of a TILLING population in R-o-18 (Stephenson et al., 2010), as reference species in micro-array design (Love et al., 2010), as well as being used in setting up a diversity fixed foundation set (DFFS) and as parents in other mapping populations. For the latter purpose, currently the genome sequences and transcript profiles of both parents are being determined (Jian Wu and Xiaowu Wang e.a., unpublished results). There are not many “immortal” *B. rapa* populations available for mapping studies, with immortal meaning that the individual lines are genetically homozygous and can thus be propagated through seeds while maintaining the established genotype in their progeny. There are few other RIL populations (Kole et al., 1997; Iniguez-Luy et al., 2009), although others may still be in development (www.brassica.info). In addition, there are several DH populations available (Zhang et al., 2006; Choi et al., 2007; Lou et al., 2007; Zhao et al., 2010; Wang et al., 2011b), which are also very useful for genetic mapping studies, although they generally comprise about half the number of recombination events compared to RIL populations and often suffer more from regions with skewedness toward one of the parental alleles.

The transgression beyond the parental lines, which was observed in the F2 generation (Bagheri et al., under review) was encouraging to produce the F7 RIL family through single-seed-descent. Out of 200 F2 lines, only 160 F7 lines were available for genotyping. This 16% loss from F2 till F7 was mostly due to plant sterility, apparently from reduced pollen production. Although this may have a genetic basis, it was not obviously related to strong skewedness of the population toward one of the two parental alleles at a particular locus. To reduce the risk of skewedness we started off with a relatively large population. Thus, most of the cross-overs between alleles located in skewed segregation region could be detected and correct linkage distances could be calculated along any skewed marker region. We did find the occasional marker with more than 75% allele skewedness toward either L58 or R-o-18 alleles, but since all of these markers were flanked by closely linked, non-skewed markers, the skewedness was found to be due to marker scoring problems rather than genetic skewedness, upon which the improperly scored markers were removed.

The residual heterozygosity in the RIL population was not significantly higher than the expected value of 1.56%. Unintended selection during single-seed-descent propagation, for instance for plant size or fecundity, could lead to increased heterozygosity at some loci (Loudet et al., 2002). Since effort was made to randomly designate which plants would be selected at each propagation cycle, we were able to avoid this type of distortion in this population. The genetic map was constructed for the F7 RILs using a mix of AFLPs, SNPs, InDel, and SSR markers. The whole genome sequence information of *B. rapa* (Cheng et al., 2011; Wang et al., 2011a) ensured the correct genome location of the SNPs, SSR, and InDel markers for which primer sequences were available. This was very efficient in resolving any mapping ambiguities and in assigning chromosome numbers to linkage groups. The current map covers a total length of 1150 cM with an average resolution of 4.3 cM. This map is comparable to two *B. rapa* reference linkage maps based on DH populations, with a total length of 1182 cM (Choi et al., 2007) and 1234.2 cM (Wang et al., 2011b), and the map reported for another RIL population, of 1125 cM (Iniguez-Luy et al., 2009). The marker resolution of 4.3 cM per marker found for this population, is also in line with the reported maps. In some cases, composite interval mapping (CIM), which is one of the QTL mapping methods we used, can be affected by an uneven distribution of markers in the genome (Zeng et al., 1999), which is why non-informative markers were omitted if they did not detect additional recombination events, to keep the smallest informative marker set. Simulation studies have shown that the advantages of increasing marker density beyond one marker every 4.3 cM are less significant than those obtained when increasing the size of the population (Darvasi and Soller, 1994; Charmet, 2000). This means that with the current marker density, there is no need to screen for additional markers in order to improve mapping efficiency.

In total 47 QTLs for 17 analyzed traits were mapped. Seed coat color is a very important trait in *Brassica* oilseed crops. A yellow seed color is known to be highly correlated with meal quality, because of the thinner seed coat, corresponding to less anti-nutritive fiber components, which is also associated with higher protein content (Tang et al., 1997; Badani et al., 2006; Wittkop et al., 2009; Liu et al., 2012). Seed coat color is a maternally inherited trait, with the alleles for black seed coat acting dominantly over the alleles for yellow seed coat. In *B. napus*, seed color is inherited in different ways, probably depending on the source of the genetic variation, and is strongly affected by environmental factors (Liu et al., 2012). Earlier studies (Stringam, 1980) proposed a two locus model for seed color in *B. rapa*, involving the *Br1* and *Br2* loci, both of which were not mapped at the time. In *B. rapa* studies involving yellow sarson oilseed types, as used in this study, a major seed color locus is found on chromosome A9 (Lou et al., 2007). In this RIL population two major QTLs were detected for seed color, *Sc1* and *Sc2*, on A9 and A3 respectively, explaining about 70% of seed coat color variation. The *Sc1* locus on A9 co-located with previously reported seed color QTLs reported for both *B. napus* and *B. rapa* (Lou et al., 2007; Liu et al., 2012; Xiao et al., 2012). Near-infrared reflectance spectroscopy measurements of acid detergent lignin (ADL) in seeds of both parental lines confirmed the expected difference in ADL corresponding to yellow and black seed (Snowdon, pers. communication) suggesting that the *B. rapa* locus we mapped to A9 affects the same gene as the A9 locus cloned from *B. napus*. This locus was found to harbor a mutation in the *CCR1* gene, encoding a cinnamoyl co-A reductase involved in lignin biosynthesis (Liu et al., 2012). In the absence of the L58 allele at *Sc1*, seeds containing the L58 allele at *Sc2* are brown, not yellow. Most of the cultivated *B. rapa* is brown-seeded, while for commercial purpose oilseed *B. rapa* with brown seeds is not preferred due to the darker coloring of the oil (Ramchiary and Lim, 2011). Introgression of the *Sc2* allele of yellow sarson types like R-o-18, could overcome this. In addition to the reported *Sc1* and *Sc2* loci, we found an additional, but very weak, *Sc3* QTL (LOD = 2.23), which mapped to A5 and accounted for 2% of the phenotypic variance. Previously, a QTL controlling yellow seed color was mapped to A5 (Teutonico and Osborn, 1994), which could concern the same locus.

Of the five QTLs detected for Ft, four Ft QTLs, *Ft1*, *Ft3*, *Ft4*, and *Ft5*, co-localized with previously mapped QTLs (Osborn et al., 1997; Lou et al., 2007; Edwards and Weinig, 2011; Lou et al., 2011). Another QTL, *Ft2* co-localized with a previously mapped, non-significant, QTL on A5 for a circadian clock parameter (Lou et al., 2011). Ft is highly co-related with plant architecture traits like Ph, Lnf, Tln, and Bn. *Ph2*, *Tln1*, and *Lnf1* co-localized with *Ft3*, while *Ft4* co-localized with *Tln4*, *Lnf4*, and *Bn1*, the only Bn QTL co-localizing with a Ft locus. Furthermore, *Ft5* co-localized with *Tln2 and Lfn3*; and finally *Ft1* co-localized with *Tln3*. *Lnf3* and *Lnf4* have been previously mapped by Lou et al. (2007), who also observed the general co-localization of *Ft* and *Lnf* loci. *Tln6* and *Lnf6* are two separate loci, mapping to A3 and A9 respectively, which did not co-localize with any *Ft* QTLs in this population, but which co-localized with *Ft* QTLs detected by Edwards and Weinig (2011).

Resistance to pod shattering is a recessive complex trait, mainly based on data from *B. napus*, which is difficult to assess because it can only be scored at maturity (Morgan et al., 2003). There are no reports related to *Brassica* loci controlling pod shattering, although work has been done on genetic engineering of pod shattering resistance, using ectopic expression of the *FRUITFULL* gene from Arabidopsis (stergaard, Kempin, Bies, Klee and Yanofsky, \O). The pod shattering QTL (*Sh*) on A9 is located in the same region as *Sc1*, but if indeed *Sc1* is caused by variation at the *CCR1* gene, as we expect, this is unlikely to be a pleiotropic effect of the same locus. Fortunately the alleles for black seeds and easy shattering are in coupling phase (Table 2), which means that selection for yellow-seeded lines could easily be accompanied by selection for improved shattering resistance. Since there is limited genetic variation for pod shattering resistance within the *B. napus* germplasm (Morgan et al., 2003), introducing pod shattering resistance alleles from *B. rapa* into a *B. napus* breeding program could well be an interesting approach.

Shattering has a significant positive correlation with the Nsps and the Sin. A significant *Sh* QTL co-located with *Nsps1*. The Nsps is also highly positively correlated with other silique related traits such as Sil and Bl. Therefore, Sil and Bl are likely to have an overall effect on silique-related traits. Sil and Bl shared one QTL, *Sil* and *Bl3* respectively. This co-localization is supported with high correlation between the two traits. Lou et al. (2007) reported two genomic regions on A1 and A7 and three loci on A5, A7, and A9, controlling Sil and Bl respectively. The *Sil* QTL on A4 reported here, is a new locus that explains 18.6% of the variance.

Vivipary (pre-harvest sprouting) is another important oilseed quality trait. A major QTL explaining 50.8% of the total variance for vivipary had previously been mapped to chromosome N11 of *B. napus* (Feng et al., 2009). We are not aware of previous work on seed vivipary QTLs in *B. rapa*. The two QTLs we detected on A9 and A6, explain about 30% of the vivipary variance. Vivipary is negatively correlated with seed oil content in our data. Also in *B. napus*, vivipary decreased seed viability and vigor and resulted in lower seed oil content (Ruan et al., 2008).

With the availability of the *Brassica rapa* genome sequence (Wang et al., 2011a) and the further development of molecular genetic tools based on the parental genotypes we used for the L58 × R-o-18 RIL population, we anticipate that the population can be a very useful additional tool to improve gene cloning approaches in *B. rapa* and thus contribute to more efficient *B. rapa* breeding.

### Conflict of interest statement

The authors declare that the research was conducted in the absence of any commercial or financial relationships that could be construed as a potential conflict of interest.
